# Can knowledge exchange support the implementation of a health-promoting schools approach? Perceived outcomes of knowledge exchange in the COMPASS study

**DOI:** 10.1186/s12889-018-5229-8

**Published:** 2018-03-13

**Authors:** Kristin M. Brown, Susan J. Elliott, Jennifer Robertson-Wilson, Michelle M. Vine, Scott T. Leatherdale

**Affiliations:** 1 University of Waterloo, 200 University Avenue West, Waterloo, Ontario N2L 3G1 Canada; 2 Department of Kinesiology & Physical Education, Wilfrid Laurier University, 75 University Avenue West, Waterloo, Ontario N2L 3C5 Canada

**Keywords:** School health, Knowledge translation, Knowledge exchange, Knowledge brokering, Qualitative research

## Abstract

**Background:**

Despite the potential population-level impact of a health-promoting schools approach, schools face challenges in implementation, indicating a gap between school health research and practice. Knowledge exchange provides an opportunity to reduce this gap; however, there has been limited evaluation of these initiatives. This research explored researchers’ and knowledge users’ perceptions of outcomes associated with a knowledge exchange initiative within COMPASS, a longitudinal study of Canadian secondary students and schools. Schools received annual tailored summaries of their students’ health behaviours and suggestions for action and were linked with knowledge brokers to support them in taking action to improve student health.

**Methods:**

Qualitative semi-structured interviews were conducted with COMPASS researchers (*n* = 13), school staff (*n* = 13), and public health stakeholders (*n* = 4) to explore their experiences with COMPASS knowledge exchange. Key issues included how knowledge users used school-specific findings, perceived outcomes of knowledge exchange, and suggestions for change.

**Results:**

Outcomes for both knowledge users and researchers were identified; interestingly, knowledge users attributed more outcomes to using school-specific findings than knowledge brokering. School and public health participants indicated school-specific findings informed their programming and planning. Importantly, knowledge exchange provided a platform for partnerships between researchers, schools, and public health units. Knowledge brokering allowed researchers to gain feedback from knowledge users to enhance the study and a better understanding of the school environment. Interestingly, COMPASS knowledge exchange outcomes aligned with Samdal and Rowling’s eight theory-driven implementation components for health-promoting schools. Hence, knowledge exchange may provide a mechanism to help schools implement a health-promoting schools approach.

**Conclusions:**

This research contributes to the limited literature regarding outcomes of knowledge brokering in public health and knowledge exchange in school health research. However, since not all schools engaged in knowledge brokering, and not all schools that engaged discussed these outcomes, further research is needed to determine the amount of engagement required for change and examine the process of COMPASS knowledge brokering to consider how to increase school engagement.

## Background

The World Health Organization defines a health-promoting school as “a school constantly strengthening its capacity as a healthy setting for living, learning and working” ([1], p. 2). A health-promoting schools approach, also referred to as Comprehensive School Health (Canada) and Coordinated School Health (United States) [2], is a whole-school approach that promotes health in school environments, through policy and community partnerships [3]. Despite the potential population-level impact of a health-promoting schools approach [4], schools face challenges regarding implementation [5, 6]. To enhance implementation, Samdal and Rowling [7] outlined eight theory-driven implementation components for health-promoting schools. *Preparing and planning for school development* describes tasks required before implementation, including identifying policies and practices to anchor the approach within the school, and establishing a team to lead implementation [7]. *Policy and institutional anchoring* involves integrating action items to target student health in school documents (e.g., school strategic plan). Both *professional development* (e.g., formal training organized by the school board) and *professional learning* (e.g., daily practices directed by school needs) are necessary to build staff capacity for adopting the health-promoting schools approach. Next, *leadership* (motivation) and *management* (logistics that allow for change) are required for organizational change and must be integrated using both *relational* (interpersonal) and *organizational* (e.g., funding and resources) *support*. *Student participation* and *partnerships* between schools and health practitioners are also critical. Lastly, in order to ensure *sustainability*, monitoring, evaluation, and continued resource allocation are required [7].

A key implementation challenge is that while the health-promoting schools approach prioritizes health, schools prioritize education [8–10], which leads to poor implementation fidelity of the health-promoting schools approach. These competing priorities align with Graham’s knowledge to action gap [11, 12], which depicts a misalignment of research and practice. Knowledge exchange, in which researchers and knowledge users collaboratively disseminate and apply research findings [13], provides an opportunity to reduce this gap. The term knowledge user describes individuals who are “likely to use research results to make informed decisions about health policies, programs, and/or practices” [14] (p. 1). Knowledge brokering is a type of knowledge exchange, in which individuals act as a link between researchers and knowledge users to support the use of research evidence in practice [13]. Despite an emphasis on knowledge translation in public health research [14–16], evaluation of these initiatives and their outcomes are still emerging [17, 18]. The need for evaluation of these strategies in school health research has also been recognized [19–21].

The Cohort Study on Obesity, Marijuana-use, Physical activity, Alcohol-use, Smoking, and Sedentary Behaviour (COMPASS) is an ongoing longitudinal study (2012–2021) of student health behaviours and secondary school environments in Ontario and Alberta, Canada [22]. Each year, students report several health behaviours in a survey, administrators report school-level policies and programs through a questionnaire, and researchers record observations of the school’s built environment [22]. In addition to traditional dissemination mechanisms (e.g., publications and presentations), two knowledge exchange strategies were integrated during the first phase (2012–2016) to support school prevention efforts to enhance student health. Each year, schools received a School Health Profile (SHP), a tailored summary of their students’ health behaviours based on survey data, including evidence-based recommendations to address student outcomes and contact information for their local public health unit [22, 23]. Each school was assigned a knowledge broker, who discussed the school’s summary and provided ongoing support as needed (e.g., identifying health priorities within the school and connecting school personnel to community agencies). While a knowledge broker contacted each school annually, the schools decided whether they engaged in knowledge brokering. Further information regarding COMPASS knowledge brokering procedures can be found online [24].

The first phase of COMPASS (2012–2016) consisted of 91 schools (n_Y1_ = 43, n_Y2_ = 89, n_Y3_ = 87, n_Y4_ = 81). As summarized in a quantitative analysis of knowledge brokering outcomes [25], Table 1 provides the levels of knowledge brokering engagement of all COMPASS schools between 2012 and 2015. Three levels of engagement were used to categorize schools. “Involved” schools were those that participated in at least one in-person meeting and/or more than one phone call with the knowledge broker annually, “somewhat involved” schools were those that participated in one phone call annually, and “not involved” schools did not engage with a knowledge broker. In the first three years of COMPASS, about half of the schools engaged in knowledge brokering (i.e., somewhat involved or involved) (Table 1).

**Table 1:** Knowledge brokering engagement levels of COMPASS schools (2012–2015)

	School Knowledge Brokering Participation Level		
Study Year	Not Involved n (%)	Somewhat Involved n (%)	Involved n (%)
2012–2013	22 (51)	12 (28)	9 (21)
2013–2014	41 (46)	38 (43)	10 (11)
2014–2015	47 (54)	30 (34)	10 (11)

*Note: numbers are provided for the first three years of COMPASS as the interviews occurred during the 2015–2016 year

COMPASS provided a case study to explore the potential impact of knowledge exchange in school health research, as well as knowledge brokering, an emerging method for which limited evaluation has been conducted [26]. This research is part of a larger convergent parallel mixed-methods study exploring the implementation and outcomes of COMPASS knowledge exchange strategies (see [25, 27]). Factors that influenced COMPASS knowledge exchange (including facilitators and challenges to knowledge brokering practice and engagement) have been examined previously [27]. This paper expands upon a quantitative analysis of knowledge brokering outcomes [25], by exploring researchers’ and knowledge users’ experiences with COMPASS knowledge exchange activities, with particular focus on perceived outcomes and suggestions for change.

## Methods

Qualitative in-depth semi-structured interviews were conducted with researchers (*n* = 13), school staff (*n* = 13), and public health stakeholders (*n* = 4) between January and October 2016, as described in [27]. Interview guides (see Additional Files) were similar for each participant group, while also capturing role differences. For example, all participants were asked about outcomes associated with knowledge user engagement in COMPASS knowledge exchange, but researchers were also prompted regarding whether there were outcomes for the research team. The COMPASS Principal Investigator notified all members of the core COMPASS team (knowledge brokers and Project Manager) and all Co-Investigators (*n* = 8) that they would be invited to participate in an interview by the first author. The first author then emailed invitations to participate.

Our school interview participants were a subset of the COMPASS (2012–2016) sample. We purposefully sampled schools engaged in knowledge brokering to varying degrees. From the COMPASS (2012–2016) sample, we identified four Ontario school boards that had at least one “involved” school (in-person meeting and/or more than one phone call with knowledge broker annually) and a mix of “somewhat involved” (one phone call annually) and “not involved” schools. Each of these boards had 4–6 schools participating in COMPASS. After gaining approval from respective school boards, we invited staff from 19 COMPASS schools for an interview via email; each school received a $30 honorarium per participant. Staff from three public health units involved in COMPASS knowledge brokering were also invited to participate via email.

All participants were provided with a letter of information detailing the research objectives and methods prior to providing consent to participate. Researchers were interviewed in person at the university (*n* = 8) or by phone (*n* = 5), while public health and school stakeholders were interviewed by phone. All interviews were conducted by the first author, a female doctoral candidate trained and experienced in qualitative methods. Interviews ranged from 20 to 90 min in duration. Eight members of the core COMPASS team (all six knowledge brokers, Principal Investigator, Project Manager) and five Co-Investigators (eight females, five males) participated, representing three Canadian universities. Six teachers, five principals, and two vice-principals (eight males, five females) from nine schools in four Ontario school boards participated. Six schools were involved in knowledge brokering, two schools were somewhat involved, and one school was not involved. Eight of thirteen school participants had engaged in knowledge brokering. The public health participants consisted of two nurses working in schools and two coordinators overseeing school initiatives within public health units. All public health participants had received SHPs for their corresponding school(s) and three had engaged in knowledge brokering. One of the public health units worked with two schools in the sample.

Interviews were audio-recorded with permission and transcribed verbatim for subsequent thematic content analysis using NVivo for Mac 11 (QSR International). Field notes were also made during the interview and used to contextualize the analysis. A template organizing style was used to code the data [28]; for each participant group, the first author read all of the transcripts to determine thematic codes (arising deductively and inductively) to compose a coding manual. The coding manuals for each participant group were used to code the respective transcripts and identify relevant data. Inter-rater reliability [29] and peer examination [30] were employed to enhance qualitative rigour of the findings. For each participant group, a second researcher coded two transcripts and the researchers’ coding of the same transcript was compared. For the knowledge broker, Co-Investigator, school, and public health transcripts, coding agreement (whether the same codes were applied to a section of text) was calculated using the methods described by Miles and Huberman [29]; agreement for all groups was greater than 70% and deemed acceptable [29]. Differences in coding were discussed and changes to the coding manual were made before coding the remaining transcripts.

Upon preliminary analysis of the results, Samdal and Rowling’s [7] eight theory-driven implementation components for health-promoting schools were chosen to explore how COMPASS knowledge exchange outcomes aligned with a health-promoting schools approach. Themes arising from the qualitative analysis were compared to, and mapped onto, Samdal and Rowling’s [7] eight components (see Discussion). Samdal and Rowling’s [7] components allowed for assessment of whether COMPASS knowledge exchange could impact a school’s readiness for implementing a health-promoting schools approach, and ultimately reduce the gap between school health research and practice.

## Results

Results are presented according to five key issues: i) feedback on the SHP, ii) how schools and public health units used COMPASS findings, iii) perceived outcomes of receiving school-specific COMPASS findings, iv) perceived outcomes of knowledge brokering, and v) suggestions for change.

### School Health Profile feedback

Knowledge users discussed the value of COMPASS findings for their schools and health units, specifically the value in school-specific, local, and longitudinal data (Table 2):

*“It’s been really useful, it’s filled a gap. We didn’t have health behaviours for youth data, there’s nowhere else we can obtain these kind of statistics, so it’s been incredibly useful for our health unit.”* (Public Health staff [PH1]).

**Table 2:** Knowledge users’ feedback regarding the School Health Profile (SHP)

	Number of Participants		
Theme	School (*n* = 13)	Public Health (*n* = 4)	Total Knowledge Users (*n* = 17)
SHP sections that participants valued	7	3	10
Year-to-year comparison	5	1	6
Gender comparison	3	2	5
Recommended interventions	2	2	4
Positive feedback about layout, content	6	2	8
Value of COMPASS findings	6	4	10
Value of school-specific and local data	4	4	8
COMPASS data perceived as equally valuable to academic data about school	3	0	3
Value of longitudinal data	1	1	2

*“The other thing is it’s a, I don’t know if the right word is, longitudinal study. So we have data over a number of years and we’re able to compare that data.”* (Principal, School 6 [S6]).

Administrators perceived COMPASS data as equally valuable to academic data about their schools:

*“It really talks about issues that affect kids’ wellbeing; kids [who] are at school, happy, not being bullied, not suffering from addiction and mental health, they’re going to be successful…. And that will affect literacy and numeracy way more, you know, than making sure that they read a series of paragraphs, right? I mean healthy kids are well-adjusted, self-actualized kids who are going to do well.”* (Principal, S7).

Knowledge users praised the layout of the SHP, finding it easy to read and understand. Participants specifically discussed the value in having i) a year-to-year comparison of student health behaviours to indicate whether, and in what direction, they were changing, ii) a gender comparison of student health behaviours, and iii) recommended interventions that schools could implement to improve student health:

*“I really appreciate the last page where you’re comparing year by year, so our first year to this year just to see, thinking back to what we may have done, what’s been successful, what’s not really made a change.”* (Teacher, S2).

*“The physical activity one was really helpful to have it broken down by gender, because we could see that girls really were far behind in the amount of physical activity, so that’s something that we did highlight to some of the schools to say, ‘there’s quite a gap here, especially for females.’”* (PH1).

*“Well just overall in the report what stands out is that you have recommendations listed, which I think is a real strength of this report. And just knowing where it comes from and that, you know, it’s evidence based.”* (PH2).

### How did knowledge users use COMPASS findings?

Seven participants read from the SHP during their interviews, indicating they used, and had access to, the resource. When asked how they used their school-specific COMPASS findings, knowledge users discussed their utility for planning purposes (e.g., School Improvement Plans, public health strategic plans, and community plans) (Table 3):

“*So we have a School Improvement Plan process… so that’s where we use this data, it gives us something to sort of ground our decision making on, and obviously we don’t use everything in the survey but we select, go through it, we analyze it, we highlight where we see a particular need*.” (Principal, S6).

**Table 3:** How knowledge users used school-specific COMPASS findings

	Number of Participants		
Theme	School (*n* = 13)	Public Health (*n* = 4)	Total Knowledge Users (n = 17)
COMPASS findings were used for:			
School planning	7	0	7
School planning documents (i.e., School Improvement Plan)	5	0	5
Grant applications	1	2	3
Public health planning documents & reports	–	2	2
Public health programming	–	2	2
Community planning documents	–	1	1
Participant shared COMPASS findings with:	11	3	14
School Staff	11	1	12
Students	6	1	7
School (parent) council	3	1	4
Public health staff	2	2	4
School board	2	0	2
Parents	2	0	2
Community groups	0	1	1

- Not relevant to participant group

Additionally, findings were used in grant applications for school- and community-based programming, and informed public health programming:

*“I think it’s given us a lot of leverage at [school 1]. Because, yes we were using the data before the big healthy eating grant, but it gave us the data we needed to be able to apply for that grant, and then we got this huge chunk of money so we’ve really been able to do a lot of activities in the last two years, which students, staff and parents saw value in, so we’re continuing to do some of those initiatives.”* (PH3).

*“One example is I was writing a briefing note on how we were going to tackle the topic of marijuana with our student population at the secondary level, and that was part of it. We included the COMPASS results from the two schools, anonymously of course*.” (PH4).

When asked who they shared the findings with, knowledge users discussed several groups including school staff, students, school (parent) councils, public health staff, school boards, parents, and community groups. All schools in the sample shared their findings with other stakeholders, although only involved schools mentioned sharing the results with students and parents:

*“I think our school council is very pleased, our trustee is very pleased, our superintendent and director are very pleased at what’s going on here…. I share every year, so again, I shared with my school council and parents this year, we put it up on our school website.”* (Principal, S1).

*“[At] parent council meetings, we pick one topic and look at those results, and discuss different ideas and what we could do to make those results better. So you know we’ve been able to use the results not only to engage students in their own health and wellbeing, staff in the students’ and their own health and wellbeing as well, because they know they’re role models. But also parents, so it’s fantastic.”* (PH3).

*“So we have been able to use the COMPASS survey results for [school name] specifically, to bring that into the conversation with the committees, to kind of highlight the fact that we do have high rates here in the community, of underage drinking. So it’s like a prevention committee made up of enforcement, school staff, the public health unit, the hospital.”* (PH1).

### Perceived outcomes of using COMPASS findings

Outcomes of using COMPASS findings were mainly discussed by knowledge users and were manifold (Table 4). All schools interviewed (across all knowledge brokering engagement levels) identified school-level outcomes of using COMPASS findings, but involved schools mentioned outcomes more frequently than less engaged schools. The most frequently mentioned outcomes were programming changes, particularly related to healthy eating, substance use, and bullying/mental health:

*“I mean we had 10% of our kids eating the recommended doses of fruits and vegetables, so that was the sole focus for 10 months of the [nutrition initiative]. So there were different fruits, different vegetables, cut up, with hummus, without hummus, in a yogurt…. So that we could hopefully maybe get the kids to like them and maybe go home and ask their parents for them, or cut them up themselves.”* (Vice principal [VP], S6).

**Table 4:** Perceived outcomes of receiving school-specific COMPASS findings

	Number of Participants			
	School (*n* = 13)	Public Health (*n* = 4)	Researchers (*n* = 13)	Total Participants (*n* = 30)
Programming changes	9	1	0	10
Healthy Eating	6	1		7
Substance use	4			4
Bullying, Mental health	3			3
Enhanced school climate, culture	4	3	7	14
Increased staff engagement and motivation for change	2	3	7	12
Increased student engagement	3	1		4
Created School Health Committee	3			3
Increased awareness of student health issues in schools	0	0	4	4
Identify health priorities to address	7	0	0	7
Health promotion and communication initiatives	4	1	0	5
Working with public health unit	4	1	0	5
Changes to physical environment	3	1	0	4
Curriculum impacts	4	0	0	4
Physical Education	3			3
Other	2			2
Prompted further investigation into findings by school	3	0	0	3

Secondly, both knowledge users and researchers described an enhanced school culture focused on health, including an increased awareness of student health in schools, motivation among school staff to make change, increased student engagement, and creating School Health Committees:

*“I have like [number] people on this [School Health] committee, I didn’t expect them all to say yes but they did. And some community members, admin, I have guidance counsellors, foods teacher, Phys. Ed. teachers, the athletic director, the health nurse from our community, the parent council chair, I’ve got five students on it, lots of people!” *(Teacher, S9)

*“I asked [school contact] some of the benefits that they’ve seen from COMPASS, and he said something along the lines of their participation providing a sense of the big picture. [School contact] said that often times within schools they’re so focused on the academic bubble… and standardized testing… and sometimes having that focus can make them forget that their job is to be looking after all aspects of students’ experience.”* (KB1).

Knowledge users also discussed identifying health priorities to address within the school, developing health promotion and communication initiatives, collaborating with public health units, and implementing physical environment and curriculum changes:

*“We do daily announcements on the TV so we have a news casting class. I had taken the information that you have given us, and picked out facts and points, so there was ‘daily tip’ on body weight or body image … and I wrote announcements for that, and they actually, I sent them the document that you guys sent me and they would flash the actual picture. Ya, so it was really neat so kids could see it.” *(Teacher, S5)

*“We had a smoking cessation program that we ran here with the [local health unit].”* (Principal, S8).

Interestingly, knowledge users mentioned the results prompted further investigation by the schools, with students from two schools conducting follow-up surveys:

*“After sharing the COMPASS report, a couple classes conducted their own surveys. So they went in to some of the elements from the COMPASS survey a little bit more deeply and asked some more probing questions of the students.”* (Principal, S2).

### Perceived outcomes of knowledge brokering

All public health staff that engaged in knowledge brokering remembered doing so. However, of the eight school staff that engaged in knowledge brokering, only five remembered doing so, and only two were familiar with, and could describe, the term “knowledge brokering”. Perceived outcomes of knowledge brokering were mainly discussed by COMPASS team researchers. Only three schools that engaged in knowledge brokering (two involved, one somewhat involved) and two public health staff discussed perceived outcomes of knowledge brokering.

Nonetheless, all participant groups described the added value that knowledge brokering offered beyond simply receiving the results (Table 5):

*“I can see the data just being put in a binder and then we’ll wait until next year. I think having that personal piece, that human piece, that contact, reflection, sharing, suggesting, meeting, again walking around the school to get a better idea of the school. I think that really kind of painted a better picture and made me commit to it, because I had some people who were committed to me.”* (Principal, S1).

**Table 5:** Perceived outcomes of knowledge brokering

	Number of Participants				
	School (*n* = 13)	Public Health (*n* = 4)	COMPASS Team (*n* = 8)	Co-Investigators (*n* = 5)	Total Participants (*n* = 30)
i) Outcomes for Knowledge Users					
Added value of knowledge brokering over SHP	3	1	7	3	14
Motivation, support for next steps	1	1	4	3	9
Access to additional data, further analyses, comparison data	1	1	5		7
Clarification of findings	2	1			3
Ideas for programming	3				3
Find out about opportunities	3				3
Relationship building	2	3	5	0	10
School-public health unit	1	1	4		6
School-researcher	0	0	3	0	3
School-level changes	1	0	7	0	8
Schools winning healthy school grants, awards	1		7		8
Changes to school facilities, new programs implemented			2		2
Increased awareness and priority of school health issues	0	0	5	0	5
Unsure if KB led to change at student-level			3		3
Unsure of long-term impacts			3		3
ii) Outcomes for COMPASS Team & Study	–	*–*	8	*–*	8
Feedback led to changes within study, will lead to future changes			5		5
Keeping schools engaged & returning year-to-year			4		4
Active involvement of graduate students in research project			4		4
Understanding implementation and context of interventions			3		3
Will incorporate knowledge brokering into future research			3		3
iii) Outcomes for Knowledge Brokers	–	*–*	7	*–*	7
Greater understanding of realities of school environment			3		3
Influenced future career prospects			3		3
Thinking about knowledge translation in own research			3		3

- Not relevant to participant group

Participants discussed the value in receiving additional survey findings (not included in the SHP) from the knowledge broker, gaining an understanding of how their students’ health behaviours compared to the rest of the schools in the sample, and receiving clarification about the findings:

*“I think it just created that opportunity to have that meeting with the school…. and then just having somebody who had more of the background on the study, and the school could ask questions, and the nurses could ask questions, so I felt that was a real strength.”* (PH2).

Further, participants mentioned the value of knowledge brokers to motivate and support schools in determining their next steps and implementing change:

*“And it seems like in some of those schools, not all of them but some of them, the knowledge broker is almost more of an impetus for them to take additional action.… we’ve had a few schools go exceptionally far beyond what we ever would have thought would be realistic for a school to want to do to try to change.”* (Principal Investigator [PI]).

Relationship building between schools and public health units, researchers, and community resources was a key outcome discussed by all participant groups:

*“I think it’s also helped them create better relationships with community partners and health units. And other projects or research surveys won’t do that. ‘Cause they don’t have anyone in place that’s all about connecting school stakeholders. So I think that’s one of the best parts about it.”* (KB4).

School-level changes were mainly mentioned by researchers, including school facilities (i.e., creating yoga studios, modifying cafeterias) and implementing new programs. During the second year of COMPASS, the provincial government offered grants for improving school nutrition and physical activity environments, and schools were able to collaborate with knowledge brokers to submit successful grants:

*“I helped with grant writing, and sending additional information, and doing some additional analyses. So a couple of my schools got pretty hefty grants, one in [city name] got [funding] to build an [nutrition initiative]. Another one of my schools got two grants, actually … to incorporate a [nutrition initiative], and then a … grant to [change physical environment to promote physical activity].” *(KB5)

However, researchers were unsure as to whether knowledge brokering would lead to changes at the student level and whether school-level impacts would be long-term:

*“…at the level of the student, I don’t know how much impact [knowledge brokering] would have had…. You kind of feel like ‘oh the school did their one week of health, did that do anything?’ Well, I mean, it got some people thinking about health for a week. You know, if they may not have before, but did that actually do anything long term? I don’t know.”* (KB2).

Additionally, the COMPASS team described positive outcomes of knowledge brokering for the study and researchers. The feedback received through knowledge brokering informed changes to the first phase of COMPASS (2012–2016) and will inform the next phase (2017–2021):

*“I think that process with the knowledge brokering has been helpful in that they’ve been getting feedback from schools and hearing ‘this is what schools really, really want, and [this is] what they’re able to do,’ kind of thing. So I think we’ve got a better idea now of what… policy/practice/environment changes are feasible, and are desirable basically, from the school standpoint.”* (Project Manager).

*“We’ve gotten very clear feedback from schools, especially through the knowledge brokers, like one of our biggest gaps is indicators related to mental health. We knew that kind of going in, we’ve got a much better picture of why we need to fill this gap moving forward.” *(PI)

Researchers attributed knowledge brokering as one of the reasons COMPASS had a low school attrition rate, with only 10 of 90 schools leaving the study over four years:

*“One of the reasons schools aren’t dropping out is that they’re recognizing that we’re really trying to do things to help advance their agenda. Answering our own research questions obviously, but also advancing their agenda. The knowledge brokers have played a big role in that. We have had some schools who’ve debated, because of competing priorities, leaving the study, and it’s often the knowledge broker interacting with them where they recognize it’s worthwhile staying in.” *(PI)

Further, through their role as knowledge brokers, graduate students were actively involved in a study where their role would normally be limited to secondary data analysis, and gained an enhanced understanding of the implementation process and context of school interventions:

*“I think it’s a great experience for students just to be able to have that interaction with schools, especially when we do school-based research. You get a better understanding of what the school environment is like, what is and is not possible given constraints on the school.”* (KB6).

Finally, knowledge brokers were exposed to various career prospects, and began thinking about knowledge exchange in their own research:

*“I’m still in the data analysis and ‘writing the thesis’ side of things, in my own research. So I haven’t quite gotten to ‘how am I going to share this information with people?’ side of things yet, but I’m definitely starting to think about it and using some stuff I’ve learned through this role.”* (KB3).

### Suggestions for change

Given that the COMPASS knowledge exchange initiatives were a pilot, we asked knowledge users and researchers for recommendations for change. Knowledge users made two suggestions for the SHP. Firstly, they would like to know what interventions other COMPASS schools have implemented:

*“But someone might be inspired, if you said, ‘like here’s some success stories about what other schools are doing with this information’. And all of a sudden, you know, you can start networking, maybe help that school connect with this school, because they said ‘ok I want to contact that person and find out what they did and how that was organized.’”* (Teacher, S4).

Despite the inclusion of provincial and national averages for health outcomes in the SHP, schools specifically wanted to know how their school compared to other COMPASS schools:

*“Comparisons I think are important because, like I see it in some of the [findings], but I think it gives you a frame of reference. A number by itself means nothing and numbers can be skewed anyway you want, but I mean, you need a frame of reference from the larger sample size to be able to assess.”* (VP, S3).

Both researchers and knowledge users discussed the need to increase understanding of the knowledge broker role among knowledge users, including the opportunity to access additional data:

*“It took me a couple years to really understand the role of the knowledge broker. So maybe initially, I could have utilized the knowledge broker a little bit more.”* (Principal, S8).

*“So I think if people that are using [the SHP] know that they can call and get more information. I think [having access to] the [survey] questions [was] really important…. so I know what I can get, right?.... I know what I can say to [knowledge broker]. ‘OK can you pull this number, can you pull that number?’ so that further helps us to do the work that we do.”* (PH3).

Both groups mentioned it would be ideal to increase opportunities for in-person knowledge brokering, and school staff discussed their preference for pre-packaged resources to aid in making changes:

*“So the easier it would be to deal with an issue that comes up in the survey, you know more support in pre-packaged things that you can give us, the more likely we would be able to implement it.”* (Teacher, S4).

Overall, knowledge users were satisfied with COMPASS knowledge exchange; six participants requested to participate in the next phase of the study without prompting:

*“I would really like to put a plug in that University of Waterloo continue with this study. I think it’s valuable that more principals in my system get on board with this…. I don’t want to give more work for the knowledge brokers, if you’re able to do that and if the funding extends, I would like to see this go for another four years.” (Principal, S2)*

## Discussion

These results expand on a quantitative analysis of knowledge brokering outcomes, which found school-level changes associated with knowledge brokering participation [25]. These qualitative results indicate the value in providing school-specific findings to participants in school health research (especially in longitudinal studies), and illustrate how the findings were used, providing a deeper understanding of the breadth of outcomes from both researcher and knowledge user perspectives. Similar to the factors influencing COMPASS knowledge exchange [27], knowledge users focused on outcomes related to their use of COMPASS findings, while researchers focused on outcomes of knowledge brokering. This raises a question as to whether engaging in knowledge brokering leads to additional outcomes for knowledge users compared to receiving school-specific findings. However, previous research suggests that determining optimal knowledge translation methods is context-dependent [31, 32]; hence, individual schools may benefit from different knowledge exchange strategies, one of which is knowledge brokering. Further, knowledge brokering may enhance the process of knowledge uptake and application in some schools, even if knowledge users do not associate it with school-level outcomes.

Outcomes for both knowledge users and researchers were identified. In addition to the value of COMPASS findings for schools, public health units gained a sense of adolescent health behaviours in their regions, which informed their programming and planning. Importantly, knowledge exchange provided a platform for partnerships between researchers, schools, and public health units. Knowledge brokering allowed researchers to gain feedback from knowledge users to enhance the study, and a better understanding of the school environment, consistent with previous research [33, 34]. For example, feedback from schools led COMPASS researchers to apply for, and receive, funding to develop a COMPASS Mental Health Module (PJT-149092). As well, knowledge brokering contributed to the retention of participating schools throughout the four-year study. However, Co-Investigators mentioned few outcomes of COMPASS knowledge exchange, as the majority were not involved with these components; considerations for the role of Co-Investigators in COMPASS knowledge exchange will be explored in future research.

Interestingly, COMPASS knowledge exchange outcomes align with factors influencing the implementation of a health-promoting schools approach [7, 35, 36]. Table 6 illustrates how COMPASS knowledge exchange activities led to achievement in each of Samdal and Rowling’s eight theory-driven implementation components for health-promoting schools [7] and incorporates opportunities to further improve the implementation of these components in the study’s next phase (2017–2021). COMPASS provides key baseline data that allow schools to assess their students’ health status, identify priorities, create action items, and establish an individual or team to lead school action. Achieving the first implementation component can be enhanced by including means for all schools in the COMPASS sample (to allow individual schools to assess how their students’ health behaviours compare) and examples of activities from other schools. Schools were able to incorporate COMPASS findings into their School Improvement Plans to identify student health priorities, integrating them with other (academic) priorities. This is a fundamental strategy for health-promoting schools [3, 37].

**Table 6:** Alignment of COMPASS knowledge exchange outcomes with Samdal and Rowling’s [7] implementation components of health-promoting schools

Implementation component [7]	Corresponding COMPASS knowledge exchange outcome	Opportunities for COMPASS knowledge exchange (next phase)
Preparing and planning for school development	Receiving school-specific findings and communicating with knowledge brokers allowed schools to: -have baseline data of student health behaviours -identify priorities related to student health (and action items) -integrate student health as a priority in their School Improvement Plans, create specific action items -establish a school contact for the COMPASS study and a School Health Committee	• For each student health outcome in the SHP, include the mean for all COMPASS schools so individual schools can understand how their results compare to other participating schools. • Include examples of interventions COMPASS schools have implemented in the SHP, to provide ideas for how to address student health outcomes.
Policy and institutional anchoring	By incorporating COMPASS findings into strategic planning documents (e.g., school improvement plans, public health unit strategic plans), knowledge users are more likely to commit to school health initiatives because school health is aligned with their organizational goals*.*	Schools could specifically incorporate the health-promoting schools approach into strategic planning documents and create school health policies.
Professional development and learning	• By providing the opportunity for schools to communicate with knowledge brokers and public health practitioners, school stakeholders can gain support in implementing school health interventions. • Opportunities for professional learning were provided when COMPASS findings were shared with school staff, which in some cases led to further health education opportunities.	Offer additional professional development opportunities related to student health behaviours and implementing school health interventions (i.e., training at the school or school board level).
Leadership and management practices	Distributed leadership was evident when school contacts shared their results with their fellow staff, public health units, and other community contacts in order to delegate action items.	Increased resource allocation (many schools have limited budgets to allocate to school health initiatives).
Relational and organizational context	• Schools used their COMPASS findings to show need for funding in grant applications, which they used to fund school health initiatives. • After receiving their COMPASS findings, schools made changes to the physical environment (e.g., new activity spaces, changes to cafeteria).	Increased resource allocation (many schools have limited budgets to allocate to school health initiatives).
Student participation	Students were involved in School Health Committees, health promotion initiatives, and collecting additional data about student health behaviors to expand upon COMPASS results.	Include examples of student-led initiatives and ways to involve students in school health in the SHP.
Partnerships and networking	• Schools shared COMPASS findings with parents and involved school (parent) council in determining action items. • Schools partnered with researchers, public health practitioners, and other community agencies to implement school health initiatives.	• COMPASS could serve as a platform to create partnerships between schools (e.g., schools sharing intervention ideas to improve student health behaviours). • Increase understanding of knowledge broker role so schools recognize that support is available. Increase in-person knowledge brokering opportunities.
Sustainability	• Annual SHPs allow schools the opportunity to monitor student health behaviours over time, assess whether school-level changes had an effect, and identify priorities. • Sustainability beyond the four-year study would be evident if knowledge users continue to incorporate student health into strategic planning documents, participate in the next phase of COMPASS, and continue to make changes to improve student health in their school.	• Increased resource allocation (many schools have limited budgets to allocate to school health initiatives). • In order to provide stronger rationale for addressing student health in schools, COMPASS could evaluate the links between school-level changes and academic outcomes.

COMPASS knowledge exchange presented opportunities for *professional learning*, as school contacts were able to communicate with knowledge brokers and public health practitioners to determine action items. *Professional learning* and *leadership and management* were reached through sharing COMPASS findings with school staff to increase awareness of student health issues and discuss possible action items. Further, principals and school champions played a key role in COMPASS knowledge exchange engagement [27]. More formal *professional development* activities such as training in school health intervention implementation could be offered [35, 36]; however, funding is limited in both school and research settings [27].

COMPASS knowledge exchange impacted both *student participation* and *partnerships*, with the inclusion of students, parents, researchers, public health, and community agencies. However, opportunities to further develop these partnerships were identified: i) COMPASS schools could form a community of practice to share ideas for addressing similar student health behaviours, ii) increase in-person knowledge brokering meetings to strengthen partnerships, and iii) increase understanding of the knowledge broker role so schools recognize that researcher support is available. Developing a community of practice for COMPASS schools would allow for knowledge transfer between knowledge users, aligning with current educator practices of sharing resources and ideas informally [37, 38]. Based on this recommendation, the COMPASS team is beginning to establish this network. While the timeframe of this research did not allow for assessing outcomes after the first four years of COMPASS, potential indicators of *sustainability* include knowledge users continuing to incorporate student health in their School Improvement Plans, making changes to improve student health, and participating in the next phase of COMPASS. Additionally, evaluating the link between school-level changes and academic outcomes would increase school buy-in for adopting a health-promoting schools approach [4, 37].

The alignment of COMPASS knowledge exchange outcomes with Samdal and Rowling’s implementation components [7] suggests that knowledge exchange in longitudinal studies may provide a mechanism for schools to implement a health-promoting schools approach. However, not all schools engaged in knowledge brokering (see Table 1, [25]), and not all schools that engaged discussed knowledge brokering outcomes, or even remembered participating in knowledge brokering. Five schools that participated in knowledge brokering did not discuss knowledge brokering outcomes; in some cases (but not all), this may be because the participant was not the main school contact who participated in knowledge brokering (i.e., the main COMPASS contact had changed over the course of the study). While all schools in the sample discussed outcomes that aligned with Samdal and Rowling’s components [7] (e.g., outcomes of using COMPASS findings), only a few achieved all components.

Further, a question arises regarding the level of knowledge exchange engagement required to achieve these implementation components. While all schools in the sample used COMPASS findings and discussed perceived outcomes of using them, more outcomes were discussed by involved schools compared to somewhat and not involved schools. This suggests our categorization of knowledge brokering engagement was meaningful; however, we found school-level outcomes for both somewhat involved and involved schools in the quantitative analysis [25], indicating further research is needed to determine the amount of engagement required for change.

Further research could investigate alternative knowledge exchange approaches to engage schools. By considering factors that influenced knowledge users’ use of study findings and knowledge brokering engagement [25, 27], we can increase research uptake and ultimately, the number of schools adopting a health-promoting schools approach. The importance of knowledge brokers reaching schools in the first year has been identified [25]; sharing case studies of how phase one schools used COMPASS findings may provide motivation for schools in the next phase to use their findings and access researcher support, enhancing subsequent outcomes. However, due to funding restrictions, COMPASS knowledge brokering may proceed differently in the second phase (2017–2021).

Only nine of the ninety-one COMPASS schools (2012–2016) were represented in this research, including only one school that did not engage in knowledge brokering. While perspectives of schools that engaged with knowledge brokering to varying degrees were included, the voice of schools that did not participate in knowledge brokering was limited in this research. It is possible that interview participants were from schools that viewed school health as a priority, and future research should make specific effort to examine schools that are less engaged. Second, we potentially missed perspectives of public health stakeholders that received the SHP but did not communicate with COMPASS researchers, since only public health personnel that communicated with COMPASS researchers were invited to participate. Nonetheless, the purpose of this study was to gain an in-depth understanding of individual experiences to expand on quantitative findings [25] and understand “what worked” in order to enhance schools’ experiences with COMPASS knowledge exchange in the next phase.

## Conclusions

This research addresses gaps in the literature related to outcomes of knowledge brokering in public health research [26] and knowledge translation in school health research [19–21]. Findings highlight the value in providing tailored summaries to schools participating in longitudinal school health research, as schools actually used these findings to make changes. Partnerships between schools, researchers, and public health were formed, leading to benefits for all groups. Knowledge brokering provided feedback to researchers to enhance the study, contributed to low school attrition, and increased researchers’ understanding of school environments. Knowledge exchange may provide a mechanism to help schools achieve the components needed for implementing a health-promoting schools approach, increasing implementation fidelity. However, further research is needed to determine the amount of engagement required for school-level changes and examine the process of knowledge brokering to consider how to increase engagement of schools. Findings from this study are being used to strengthen knowledge exchange in the next phase of COMPASS and can also inform similar activities in school health and public health research.

## Abbreviations

KB: Knowledge broker
PH: Public health staff
PI: Principal Investigator
SHP: School Health Profile
VP: Vice principal
